# Distinct Patterns in Human Milk Microbiota and Fatty Acid Profiles Across Specific Geographic Locations

**DOI:** 10.3389/fmicb.2016.01619

**Published:** 2016-10-13

**Authors:** Himanshu Kumar, Elloise du Toit, Amruta Kulkarni, Juhani Aakko, Kaisa M. Linderborg, Yumei Zhang, Mark P. Nicol, Erika Isolauri, Baoru Yang, Maria C. Collado, Seppo Salminen

**Affiliations:** ^1^Functional Foods Forum, Faculty of Medicine, University of TurkuTurku, Finland; ^2^Division of Medical Microbiology, Department of Pathology, University of Cape TownCape Town, South Africa; ^3^Food Chemistry and Food Development, Department of Biochemistry, University of TurkuTurku, Finland; ^4^Department of Nutrition and Food Hygiene, School of Public Health, Peking UniversityBeijing, China; ^5^Institute of Infectious Disease and Molecular Medicine, University of Cape TownCape Town, South Africa; ^6^National Health Laboratory Service of South Africa, Groote Schuur HospitalCape Town, South Africa; ^7^Department of Pediatrics, University of TurkuTurku, Finland; ^8^Institute of Agrochemistry and Food Technology, National Research Council (IATA-CSIC)Valencia, Spain

**Keywords:** microbiome, human milk, fatty acids, geography, delivery

## Abstract

Breast feeding results in long term health benefits in the prevention of communicable and non-communicable diseases at both individual and population levels. Geographical location directly impacts the composition of breast milk including microbiota and lipids. The aim of this study was to investigate the influence of geographical location, i.e., Europe (Spain and Finland), Africa (South Africa), and Asia (China), on breast milk microbiota and lipid composition in samples obtained from healthy mothers after the 1 month of lactation. Altogether, 80 women (20 from each country) participated in the study, with equal number of women who delivered by vaginal or cesarean section from each country. Lipid composition particularly that of polyunsaturated fatty acids differed between the countries, with the highest amount of n-6 PUFA (25.6%) observed in the milk of Chinese women. Milk microbiota composition also differed significantly between the countries (*p* = 0.002). Among vaginally delivered women, Spanish women had highest amount of Bacteroidetes (mean relative abundance of 3.75) whereas Chinese women had highest amount of Actinobacteria (mean relative abundance 5.7). Women who had had a cesarean section had higher amount of Proteobacteria as observed in the milk of the Spanish and South African women. Interestingly, the Spanish and South African women had significantly higher bacterial genes mapped to lipid, amino acid and carbohydrate metabolism (*p* < 0.05). Association of the lipid profile with the microbiota revealed that monounsaturated fatty acids (MUFA) were negatively associated with Proteobacteria (*r* = -0.43, *p* < 0.05), while *Lactobacillus* genus was associated with MUFA (*r* = -0.23, *p* = 0.04). These findings reveal that the milk microbiota and lipid composition exhibit differences based on geographical locations in addition to the differences observed due to the mode of delivery.

## Introduction

Breastfeeding has an influential role in the increasing burden of non-communicable disease, including allergy and obesity (Aaltonen et al., 2011; Kelishadi and Farajian, 2014). Not only does it provide complete personalized nutrition to infants and is important for optimal growth and development, but it also confers immunological protection during critical period in life. A delicate balance of stimulatory, even inflammatory, maturational signals, together with myriad of anti-inflammatory compounds is transferred from mothers to infant via breastfeeding. The most potent immunoregulatory factors include cytokines, growth factors, specific proteins such as lactoferrin, peptides, fatty acids, human milk oligosaccharides, and microbes (Collado et al., 2012).

Breastfeeding mothers, however, do not comprise a uniform group. Maternal environmental conditions such as dietary habits and lifestyle, individual and circadian variations in fatty acid synthesis, genetic factors, and lactation time, all influence the composition of breast milk (Boersma et al., 1991; Brenna et al., 2007; Mäkelä et al., 2013). Recent reports have suggested antibiotics, maternal health and gestational age as factors impacting milk microbiota (Hunt et al., 2011; Cabrera-Rubio et al., 2012, 2016; Khodayar-Pardo et al., 2014; Soto et al., 2014; Hoashi et al., 2015). Intriguingly, mode of delivery is also known to affect breast milk microbiota composition (Collado et al., 2012; Khodayar-Pardo et al., 2014; Cabrera-Rubio et al., 2016).

Since the composition of breast milk shows marked individual variation, this may affect the immunoregulatory properties of breast milk. However, population or region-specific factors influencing microbiota composition are largely unexplored. Similarly, the potential relationship of milk microbiota with other milk compounds such as lipids has not been well established.

As personalized breast feeding provides the exclusive nutrition to an infant guiding the development of the gut microbiota and maturation of the immune system, we aimed to identify the impact of four different geographical locations: Asia, Africa, and North and South Europe on breast milk composition. We focused on the microbiome and the fatty acid composition and the impact of mode of delivery on breast milk composition across those locations.

## Materials and Methods

### Breast Milk Sample Collection

The study group comprised 80 healthy women volunteers from different geographical locations representing urban lifestyles including China (Beijing area), South Africa (Cape Town), Finland (southwestern area), and Spain (Valencia, Mediterranean area). Subjects from each country (*n* = 20) were grouped according to the mode of delivery, vaginal (*n* = 10) and cesarean section (*n* = 10).

Maternal characteristics such as age, weight, body mass index (BMI) and parity were collected at the time of enrolment. All participants received detailed information about the study, written informed consent was obtained and the study protocol was approved by the Ethics Committees of the respective participating institutions, Spain (Bioethics Committee of CSIC and from the Regional Ethics Committee for Biomedical Research), Finland (Turku University Hospital), China (Medical Research Board of Peking University), and South Africa (University of Cape Town, Human Research Ethics Committee).

Before sample collection, the mothers were given oral and written instructions for standardized collection of samples. The mature milk samples (1 month post-partum) were collected manually into a sterile tube. Prior to collection, nipples and mammary areola were cleaned with soap and sterile water and soaked with chlorhexidine to reduce contamination by skin flora. The first drops of milk (approximately 500 μL) were discarded. All the samples were kept frozen at -20°C until delivery to the laboratory and then stored at -80°C until further analysis. All the samples were shipped to Finland for storage, processing, lipid analysis, and DNA extraction as a part of collaborative project.

### Extraction of Lipids and Isolation of Triacylglycerols (TAGs) and Phospholipids (PLs)

An internal standard mixture of triheptadecanoin (Sigma–Aldrich, St.Louis, MO, USA) and dinonadecanoylphosphatidyl choline (Sigma–Aldrich, St.Louis, MO, USA) was added to the thawed milk (from 178 to 539 mg). Then 1.5 mL methanol, 3 mL chloroform and 0.8 mL 0.88% KCl in water were added and the blend was thoroughly vortexed. The tubes were centrifuged 2000 × *g* for 3 min to separate the layers, and the chloroform rich layer was collected, evaporated to dryness, and re-suspended in chloroform (Folch et al., 1957). The triacylglycerols (TAG) and phospholipids were isolated from the extracted lipid mixture with solid phase extraction based on silica columns as described previously (Hamilton and Comai, 1988).

### Preparation of Fatty Acid Methyl Esters and Their Chromatographic Analysis

Fatty acid methyl esters (FAME) were prepared with the sodium methoxide method (Christie, 1982; Hamilton and Comai, 1988). In short, the lipids were suspended in 1 mL dry diethylether; then 25 μL methylacetate and 25 μL sodium methoxide were added, and the blend was incubated for 5 min with shaking. The reaction was stopped with 6 μL of acetic acid. The tubes were centrifuged 2000 × *g* for 5 min, after which the supernatant was collected and gently evaporated to dryness, and the resulting FAME were dissolved in hexane. The FAME were analyzed with gas chromatography (Shimadzu GC-2010 equipped with AOC-20i auto injector, flame ionization detector, Shimadzu corporation, Kyoto, Japan). A wall-coated open tubular column DB-23 (60 m × 0.25 mm i.d., liquid film thickness 0.25 μm, Agilent technologies, J.W. Scientific, Santa Clara, CA, USA) was used for the analysis. Helium was used as the carrier gas. Splitless/split injection was used, and the split was opened after 1 min. The injection volume was 0.5 μL, and inlet temperature 270°C. The initial oven temperature, which was held for 1 min was 130°C. The oven temperature was programmed to rise at a rate of 4.5°C/min to 170°C and 10°C/min to 220°C, where it was held for 3.5 min, and further at 10°C/min to 230°C and 60°C/min to 240°C, where it was held for 7 min. The detector temperature was 280°C. Peaks were identified by comparison of their retention times to the retention times of known external standard mixtures, Supelco 37 Component FAME Mix (Supelco, St. Louis, MO, USA), 68D (Nu-Check-Prep, Elysian, MN, USA), GLC-11A (NuCheck Prep, Elysian, MN, USA), and GLC-490 (Nu-Check-Prep, Elysian, MN, USA), and quantified in relation to the internal standards and corrected with response factors calculated based on analysis of standard mixtures. The identified fatty acids, i.e., TAG and PLs, were (grouped for further analysis) classified as saturated fatty acids (SAFA), monounsaturated fatty acids (MUFA) and polyunsaturated fatty acids (PUFA), and PUFA further sub-classified into n-3 and n-6 PUFAs.

### Microbial DNA Extraction and Sequencing

Breast milk samples were centrifuged at 14,000 rpm for 20 min at 4°C, fat was removed and the pellet was used for total DNA extraction. Bead beating was carried out using FastPrep^®^ (FP120-230, Bio 101 ThermoSavant, Holbrook, NY), and DNA extracted from the supernatant using the InviMag^®^ Stool DNA kit (Stratec Molecular, Berlin, Germany) with the KingFisher magnetic particle processor (Thermo Fisher Scientific Oy, Vantaa, Finland). Purified total genomic DNA was shipped to South Africa for PCR and sequencing. PCR amplification and sequencing was carried out at the Centre for Proteomic and Genomic Research (Cape Town, South Africa) using Illumina MiSeq sequencing platform. Primers targeting hypervariable V4 region of the bacterial 16S rRNA gene were used (bacterial/archaea primers 515F and 806R) according to previously described methods and modified for the Illumina MiSeq platform. Controls included two no template controls (NTC) and two NTC spiked with 6 different *Staphylococcus* species at 20 ng/uL each. Sequencing data has been submitted to NCBI with SRA accession: SRP082263 and submission ID: SUB1772296.

### Microbial Community Analysis

Quality assessment of obtained reads was done using prinseq-lite program (Schmieder and Edwards, 2011) with defined parameters (i.e., min_length:50, trim_qual_right:20, trim_qual_type:mean, trim_qual_window:20). Filtered and demultiplexed sequences were processed using the open-source software QIIME (with default parameters; Caporaso et al., 2010, 2012). A total of 80 samples were sequenced, with mean of 11,538 sequences per sample (500 minimum sequences per sample). One of the Finnish samples generated fewer than 500 sequences after quality filtering and was excluded from further analysis. The remaining sequences were then binned into Operational Taxonomic Units (OTUs) using *de novo* OTU picking based on 97% identity using the August 2013 build from Greengenes reference database. Alpha diversity was determined from rarefied tables using indices which includes Shannon-Wiener index, Chao 1 index for richness and Observed Species (number of unique OTUs) and Phylogenetic Distance (PD_whole) were also determined. Beta diversity was computed with OTU table using UniFrac. Weighted and unweighted unifrac distance along with sample metadata was used as input for principal coordinate analysis (PCoA). OTU table was rarefied to 500 sequences per sample for all samples, in computing diversity measures to avoid variations in sequencing depth. Calypso version 5.2^1^ was used with data transformed by centered log ratio with total sum normalization, to generate Venn diagram for shared phylotypes at family level, and Redundancy Analysis (RDA) plot for multivariate analysis using OTU phylotypes, and country as factor.

### Metagenomic Prediction

Functional metagenomes were predicted from 16S rRNA reads using PICRUST (Langille et al., 2013). Briefly, OTU’s based on the closed reference OTU picking method were selected using QIIME v 1.9.1 and by querying the data against a reference database (GreenGenes database v 13_8); OTUs were assigned at 97% identity. The resulting OTU table was used to predict metagenomes, which were functionally categorized based on KEGG (Kyoto Encyclopedia of Genes and Genomes) pathways at different levels of classification. The data was analysed using galaxy interface^2^ for LEfSe [Linear Discriminant Analysis (LDA) Effect Size] to elicit differential microbial functional pathways in different samples. The differential abundant features are ranked by effect size after LDA with effect size threshold between 2 and 3 (on a log_10_ scale).

### Statistical Analysis

Relative abundance of fatty acids in TAG and phospholipids were statistically tested using two-factor analysis of variance followed by Tukey’s HSD *post hoc* test to compare the main effects of country of origin and mode of delivery; and the interaction effect between country of origin and mode of delivery. Microbial group significance based on phylotypes was compared at different levels by ANOVA based on the QIIME pipeline. Pearson’s correlation matrix was calculated for the gut microbiota (phylum and genus level) and the TAG and phospholipids. Heatmaps of correlation coefficients and principal component analysis were constructed using the g-plots and factoextra package in R (R Core Team, 2010). The statistical analysis was performed with R statistical environment version 3.1.1.

## Results

### Subject Selection

The mean age of the women from the four countries (*n* = 80) varied from 32 to 34, with mean pre-gestational BMI of 24–25 (normal weight), except the Chinese group with mean BMI of 21.7 (SD ± 1.9) (normal weight, because of Asian origin) (*p* < 0.05). All women recruited in the study, intended to breastfeed irrespective of the mode of delivery. Interestingly, the women, who delivered vaginally, had mean BMI of 24 (SD ± 2.6), except the Chinese group with mean BMI of 21.9 (SD ± 1.5). South African and Finnish women, who delivered by cesarean section had mean BMI of 26. All women, who delivered via C-section, were given prophylactic antibiotics, except Finnish women where no prophylaxis is routinely used as per the hospital policy. All South African women got cefazolin before C-section whereas Spanish women either got cefazolins or penicillin related. Chinese women who delivered by C-section either got metronidazole, cephalosporins or azithromycin.

### Breast Milk Fatty Acid Profile Based on Phospholipids and Triacylglycerols

Our results showed that the fatty acid profile of phospholipids varied across the four countries with significant differences in the levels of total PUFA (*p* < 0.001), n-6 PUFA (*p* < 0.001), n-3 PUFA (*p* = 0.003), and SAFA (*p* < 0.001). Interestingly, north and south of Europe, i.e., Spain and Finland differed significantly in the n-3 (*p* < 0.001) and n-6 (*p* = 0.007) fatty acid levels in TAG.

**FIGURE 1:**
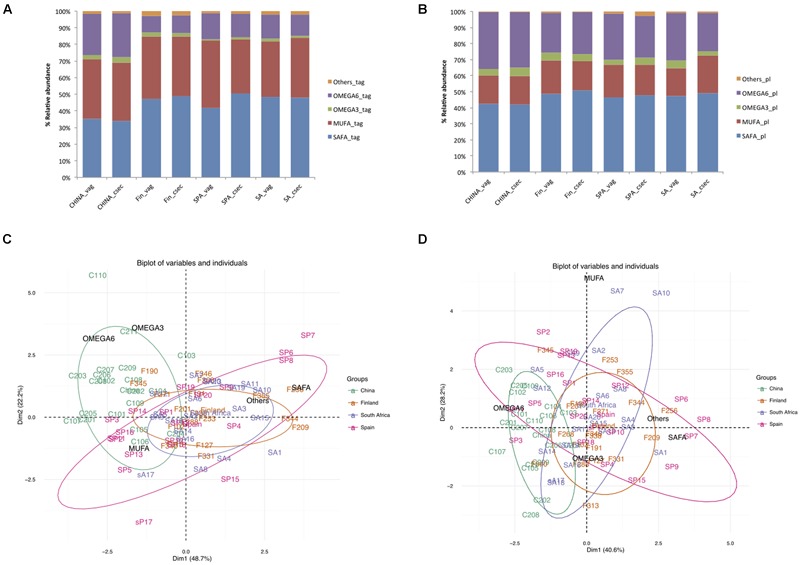
**Comparison of percentage relative abundances of fatty acids taking mode of delivery and country as factors **(A)** Triacyglycerols **(B)** Phospholipids; **(C)** PCA of triacyglycerols **(D)** PCA of phospholipids, ellipses in the plot represent 95% confidence interval of grouping based on countries**.

In China, the levels of SAFAs (34.7% of total TAG) in TAG were lower (*p* < 0.001) than in the other areas, with relative order corresponding to (Finland >South Africa >Spain) (Supplementary Table 1). The Chinese and Finnish samples had relatively higher n-3 PUFA than the Spanish and South African samples. On the other hand, n-6 PUFA was found to be highest (34.84% in PL, 25.66% in TAG) in Chinese samples (**Figures 1A,B**). These differences observed in TAG and phospholipids in the Chinese samples were also revealed by PCA, which showed distinct Chinese clustering compared to the samples from other areas (**Figures 1C,D**).

The impact of the mode of delivery is not consistent across the countries. However, fatty acid profile differed between the countries as the South African women who delivered by cesarean section had higher MUFA (*p* < 0.001) in their milk than those women who delivered by the vaginal delivery; whereas Spanish women who delivered by vaginal delivery had higher MUFA in TAG (*p* = 0.003) (**Table 1**).

**Table 1:** Relative abundancies (%) of fatty acids in phospholipids and triglycerides presented as predicted marginal means with 95% confidence intervals.

	China		Finland		South Africa		Spain				
	
	Csec (*n* = 20)	Vaginal (*n* = 20)	Csec (*n* = 19)	Vaginal (*n* = 20)	Csec (*n* = 20)	Vaginal (*n* = 20)	Csec (*n* = 20)	Vaginal (*n* = 20)	p (Country)	*p* (Delivery)	*P* (Country^∗^ delivery)
PL SAFA	42.05 (39.69–44.41)	42.37 (40.01–44.72)	51.05 (48.69–53.41)	48.33 (45.84–50.81)	49.33 (46.98–51.69)	49.33 (46.98–51.69)	47.79 (45.43–50.14)	46.48 (44.12–48.84)	<0.001	0.089	0.61
PL MUFA	17.81 (15.60–20.01)	17.83 (15.62–20.03)	17.91 (15.70–20.12)	23.46 (21.25–25.66)	23.46 (21.25–25.67)	17.55 (15.34–19.75)	19.13 (16.92–21.34)	20.18 (17.98–22.39)	0.11	0.50	**0.001**
PL OMEGA3	5.04 (4.27–5.82)	3.98 (3.21–4.76)	4.61 (3.84–5.39)	4.97 (4.15–5.79)	2.31 (1.53–3.08)	4.58 (3.80–5.35)	4.31 (3.54–5.09)	3.16 (2.39–3.94)	0.003	0.72	**<0.001**
PL OMEGA6	34.50 (32.56–36.44)	35.18 (33.25–37.12)	25.79 (23.86–27.73)	25.22 (23.18–27.27)	23.92 (21.99–25.86)	29.99 (28.06–31.93)	26.19 (24.25–28.12)	28.88 (26.95–30.82)	**<0.001**	**0.002**	**0.007**
PL PUFA	39.55 (37.68–41.41)	39.17 (37.30–41.03)	30.40 (28.53–32.27)	30.20 (28.23–32.16)	26.23 (24.36–28.10)	34.57 (32.70–36.44)	30.50 (28.63–32.37)	32.05 (30.18–33.92)	**<0.001**	**<0.001**	**<0.001**
PL Others	0.60 (0.11–1.09)	0.64 (0.15–1.13)	0.64 (0.15–1.13)	0.69 (0.18–1.21)	0.98 (0.49–1.47)	0.70 (0.22–1.20)	2.58 (2.09–3.07)	1.29 (0.80–1.78)	**<0.001**	**0.038**	**0.026**
TAG SAFA	34.06 (29.47–38.71)	35.35 (30.7–40.00)	48.95 (44.30–53.60)	47.40 (42.5–52.30)	48.16 (43.50–52.61)	48.46 (43.81–53.12)	50.42 (45.77–55.07)	41.8 (37.15–46.45)	**<0.001**	0.20	0.15
TAG MUFA	34.96 (31.42–38.50)	35.90 (32.36–39.44)	35.67 (32.12–39.21)	37.54 (33.80–41.27)	35.81 (32.26–39.35)	33.56 (30.12–37.10)	32.70 (29.16–36.24)	40.56 (34.02–44.10)	0.65	0.010	**0.045**
TAG OMEGA3	3.16 (2.61–3.71)	2.26 (1.71–2.81)	2.37 (1.82–2.91)	2.48 (1.90–3.06)	1.36 (0.81–1.91)	1.53 (0.98–2.08)	1.16 (0.61–1.70)	0.87 (0.32–1.42)	**<0.001**	0.25	0.20
TAG OMEGA6	26.41 (23.89–28.93)	24.91 (22.39–27.43)	10.52 (8.00–13.04)	10.10 (7.44–12.75)	12.49 (9.97–15.01)	14.33 (11.81–16.85)	13.96 (11.44–16.48)	15.38 (12.86–17.90)	**<0.001**	0.70	0.52
TAG PUFA	29.57 (26.90–32.24)	27.17 (24.50–29.85)	12.89 (10.22–15.56)	12.58 (9.76–15.39)	13.85 (11.17–16.52)	15.86 (13.19–18.53)	15.12 (12.44–17.79)	16.25 (13.58–18.93)	**<0.001**	0.90	0.38
TAG Others	1.41 (1.05–1.76)	1.58 (1.23–1.93)	2.49 (2.14–2.84)	2.49 (2.11–2.85)	2.19 (1.84–2.54)	2.11 (1.77–2.47)	1.76 (1.41–2.11)	1.38 (1.03–1.74)	**<0.001**	0.58	0.48

*Significance levels were determined by two-way analysis of variance testing the effect of country, mode of delivery and their interaction. Differences between factors were determined by Tukey HSD *post hoc* comparisons. Bolded *p*-values: significant differences.*

### Microbiota Compositional Analysis

**FIGURE 2:**
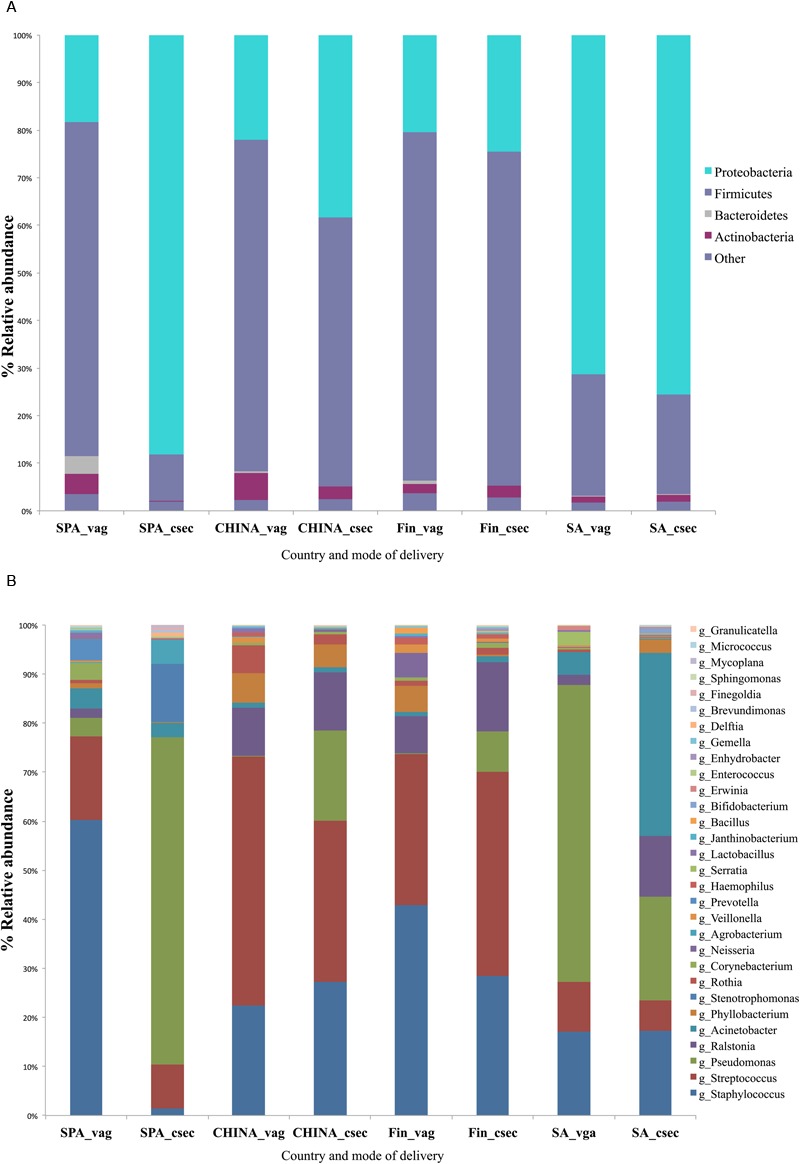
**Comparison of percentage relative abundances taking mode of delivery and country as factor at **(A)** Phyla level (top 5 phylotypes based on relative abundance), **(B)** Genus level (top 30 genera based on total relative abundance)**.

Overall, the sequencing resulted in 911,547 clean and filtered sequences with mean count of 11,538 (±9,025 SD) reads per sample. Milk microbiota composition differed significantly across all the countries (ANOSIM test, *p* = 0.002). However, Finnish and South African samples had relatively similar profiles for vaginal and cesarean delivery (**Figure 2A**). Spanish women who delivered vaginally had the highest level of Bacteroidetes in breast milk in comparison to women from other countries. In general, South African women had significantly higher relative abundance of Proteobacteria (*p* = 0.004) when compared to other countries. This could have been attributed to higher level of *Enterobacteriaceae* and *Pseudomonadaceae* family observed in the milk samples of the South African and Spanish women (*p* < 0.05). The samples from Finland had higher levels of Firmicutes and lower levels of Proteobacteria when compared to the samples from other countries (*p* = 0.004). Among the mothers with vaginal delivery, the Chinese women had the highest level of Actinobacteria. At genus level, Chinese women had higher levels of *Streptococcus*, and Spanish women higher levels of *Propionibacterium* and *Pseudomonas* in Spanish when compared to women from all countries with mode of delivery as factor (*p* < 0.05) (**Figure 2B**).

Spanish mothers who delivered by cesarean section had decreased alpha-diversity (*p* = 0.013) and hallmarked by higher level of Proteobacteria in comparison to all women from other countries. Overall, other diversity indices across all the study regions were similar. Within the Spanish group, women who delivered vaginally had significantly higher diversity (*p* = 0.021) when compared to those who delivered by C-section.

**FIGURE 3:**
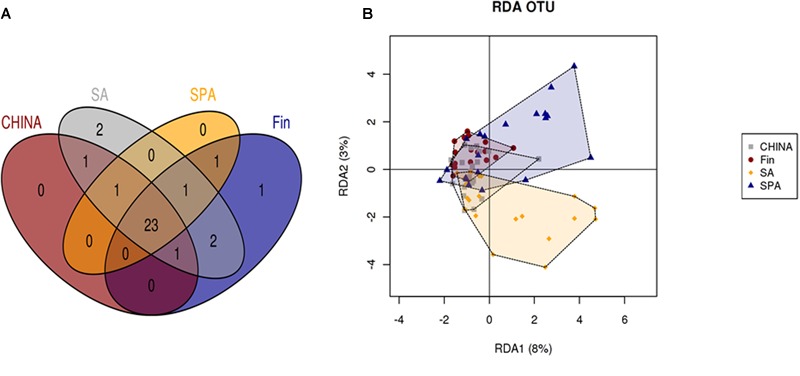
**(A)** Shared phylotypes at family level across countries. **(B)** RDA analysis at OTU level, across different countries.

Overall microbial community at OTU level was significantly different in all countries suggesting a geographical area-dependent microbiota. The European areas (Spain and Finland) had significant differences in microbial communities even when mode of delivery was taken into account (*p* < 0.05). RDA analysis at OTU level showed that the microbiota profile of Spanish and South African milk samples were more diverse when compared to those of Finnish and Chinese women who formed tight clusters (**Figure 3B**). Due to the impact of individual variability and geographical area, we identified a core microbiome (at family level) with 23 phylotypes that constituted the core milk microbiome across all the countries (**Figure 3A**). *Lactobacillaceae* was found uniquely in samples from Finland, *Bifidobacteriaceae* found only in South African women and *Enterococcaceae* in samples from all the countries except China (Supplementary Table 2).

### Imputed Metagenomic Analysis and Metabolic Pathway Prediction

**FIGURE 4:**
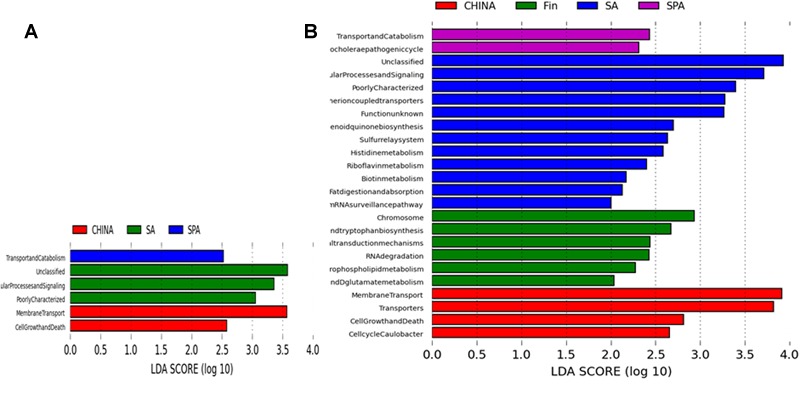
**PICRUSt based predicted metagenome using KEGG annotation at levels **(A)** L2 **(B)** L3, representing LDA scores for differentially abundant genes/pathways (Log LDA = 2) across different countries**.

Metagenomic prediction based on PICRUSt analysis and LEfSe results revealed siginificant difference in abundance of KEGG pathways in South African, Spanish and Chinese women at L2 of KEGG pathway (**Figure 4**). South African samples were differentially mapped to metabolic pathways as predicted by KEGG based on annotation at L2 and L3 levels (**Figure 4**). Some of the major differential pathways included fat digestion and absorption, biotin metabolism and histidine metabolism. Spain and South African women had significantly higher while Finnish women had significantly lower number of reads mapped to lipid metabolism, amino acid metabolism and carbohydrate metabolism when compared among all the countries (*p* < 0.05).

### Association of BMI, Fatty Acid Profile with Microbiota Composition

Redundancy analysis at OTU level, taking all the fatty acid as factors, revealed that geographical location (*p* = 0.003) is one of major contributing factor associated with differences in microbiota observed in all women. Moreover, the fatty acids, SAFA of TAG (*p* = 0.002) and PUFA of phospholipids (*p* = 0.05) also found to be contributing factor associated with the differences observed in microbiota.

**FIGURE 5:**
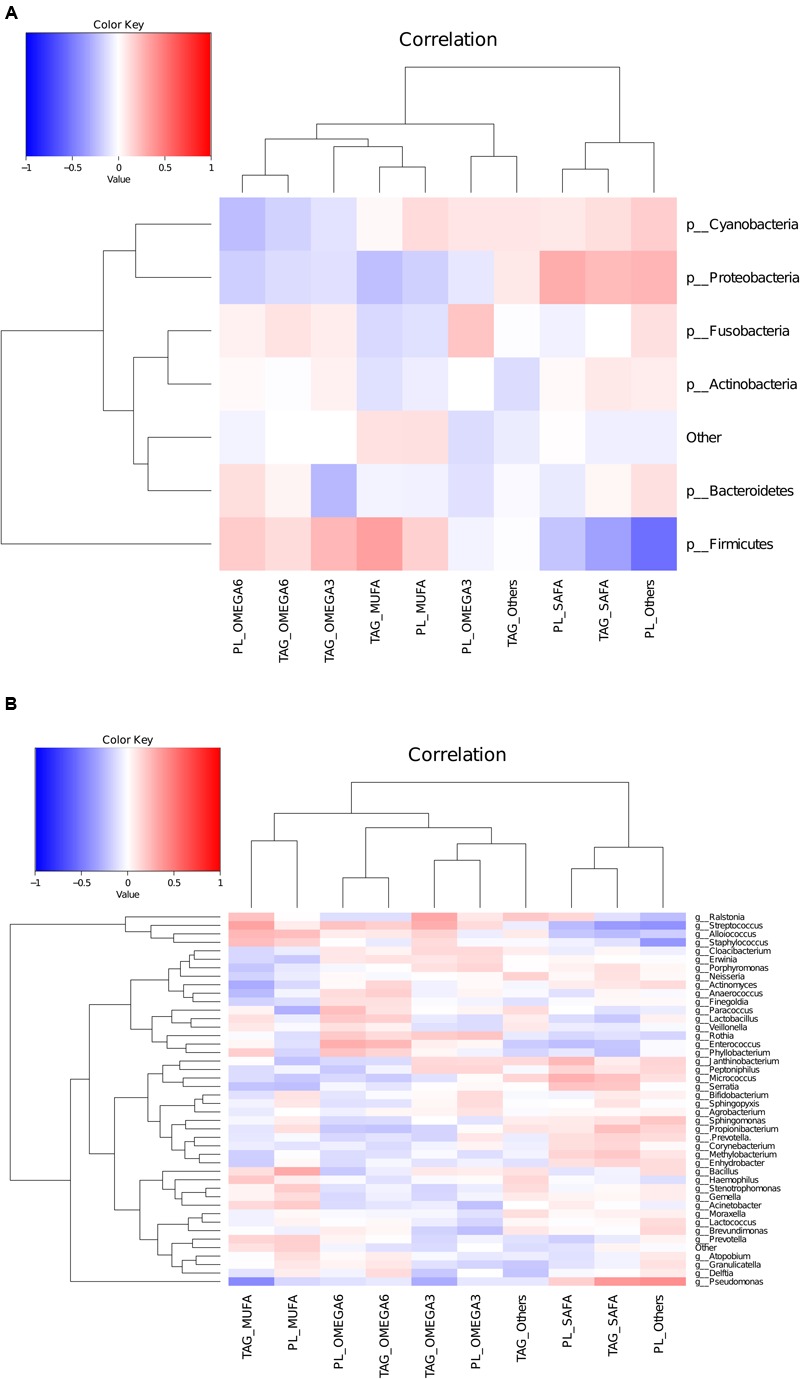
**Heatmap to show correlation between lipidomic data and microbiota composition at phylum level **(A)** and genus level (B)**.

Regression analysis at phyla level, taking BMI as a factor, showed that in more than 98% of women (irrespective of their country) Firmicutes was positively associated with BMI. In all samples, MUFA in TAG were found to be negatively associated with Proteobacteria (*r* = –0.43, *p* < 0.05). In more than 90% of participating women, MUFA and PUFA in TAG were found to have a significant positive association while SAFA in triacylglycerols was negatively associated, with the *Streptococcus* and *Acinetobacter* genus. SAFA (in both TAG and phospholipids) was also found to be positively associated with *Pseudomonas*. In phospholipids, *Lactobacillus* genus was negatively associated with MUFA (*r* = –0.23, *p* = 0.04) and n-3 PUFA was negatively associated with *Bifidobacterium* genus (*r* = –0.26, *p* = 0.03). The heat map generated from Pearson correlation analysis based on microbiota at genus level with major categories of TAG and phospholipids showed that higher abundance of *Pseudomonas* was correlated with lower levels of MUFA in TAG, whereas higher abundance of *Streptococcus* genus were correlated with lower levels of SAFA in TAG. Within TAG, *Staphylococcus* genus was found to be negatively correlated with MUFA (**Figure 5**).

## Discussion

Breastfeeding is one of the most important practices, which is recommended worldwide for the adequate nutrition and health of newborns. We reported the effect of environment (geographical area and/or lifestyle) in the milk microbiota composition, lipids and the relationship between them. These factors are transferred to infant during breast-feeding and will likely create a hallmark in infant gut microbiome and immune system maturation. All the milk samples were obtained with the same protocol (to avoid bias) and processed in Finland. Sequencing was carried out at South Africa, to avoid effect of different batches (if processed at their respective locations).

Breast milk fat is a major source of energy, providing over 40% of the total energy for infants during first 6 months of life. Fat globules in breast milk consist of TAG (98–99% of milk lipids) and small amount of monoacylglycerols, diacylglycerols, free fatty acids surrounded by milk fat membrane of different phospholipids (Delplanque et al., 2015). Some of the indispensable lipid components include PUFAs and long chain PUFAs (LC-PUFA) of the n-6 and n-3 series and lipid soluble vitamins. A recent study reported complex interactions between fatty acids and protein profiles which were influenced by lactation stage and also, gestational age (Collado et al., 2015). Our results demonstrated differences in human milk fat composition and fatty acid profiles of study milks between the four countries. The highest variability was observed in n-6 PUFA in TAG fatty acids and in phospholipids. In general, human milk fat content exhibits high degree of inter-individual variability, with maternal nutrition, adiposity and health being some of the important factors. This is also confirmed by two independent studies, in a Danish study which involved 244 women revealed that fat content varied from 1.8 g/dl at lower 2.5 percentile to 8.9 g/dl at the upper 97.5 percentile, and in another study based on women from United States the range varied from 0.7 g/dl to 7.06 g/dl (Robinson and Caplan, 2015). The variations observed in Chinese population, could be attributed to number of factors majorly to maternal nutrition.

Recent studies have reported the high diversity of microbial composition in human milk (Hunt et al., 2011; Cabrera-Rubio et al., 2012, 2016; Fernandez et al., 2013; Jost et al., 2013). We also confirmed previous findings as those reported in the Finnish population (Cabrera-Rubio et al., 2012) and Spanish population (Cabrera-Rubio et al., 2016), that mode of delivery associates with milk microbiome but the impact differed depending on the country. In addition to the differences observed in milk microbiome, another recent report in USA has shown differences in glycolysation pattern based on mode of delivery (Hoashi et al., 2015). However, another study on milk microbiota of 39 Canadian women could not find any difference based on mode of birth, which could be also because of high number (*n* = 28) of preterm deliveries (Urbaniak et al., 2016). It is also well-established that genetics and environmental factors influence gut microbiota composition. Big worldwide cohorts —MetaHIT (European), HMP (American), and Chinese diabetes cohorts—reported that the inter-country variation in gut microbiota composition significantly exceeded inter-personal variation (Lloyd-Price et al., 2016). A study targeting gut microbiota from five European countries (Sweden, United Kingdom, Germany, Italy, and Spain), representing varied life style characteristics, showed that higher proportion of bifidobacteria was found in Northern European countries while *Bacteroides* was more prominent in southern countries (Fallani et al., 2010). Interestingly, we also found in this study that the breast milk samples from Spanish women exhibited higher diversity than those from northern Europe; although our study is focused on breast milk which may in turn affect the infant gut microbiota. Despite the great inter-individual variability, accumulating data show that breast milk microbiota is dominated by Staphylococci, Streptococci, specific Proteobacteria group, and *Propionibacteria* (Hunt et al., 2011). Additionaly, specific anaerobic bacterial groups such as *Bifidobacterium, Bacteroides, Parabacteroides* and members of Clostridia have been also detected as common constituents of breast milk (Sinkiewicz and Ljunggren, 2008; Ted et al., 2014).

Overall, metagenomic prediction revealed that some of the genes associated with lipid, amino acid, and carbohydrate metabolism were predominated in South African and Spanish women, which was also observed in overall microbial community similarity. These variations in pathways could also be due to differences in major metabolites present in milk and their interaction with microbes. However, transcriptome profile of human breast milk also suggested that the dietary modifications due to fat intake was not reflected in expression of genes associated with fatty acid uptake/synthesis or cell-cycle regulation (Yahvah et al., 2015). Microbiota and fatty acid compositional analysis revealed that the geographic origin is a more important factor for the observed differences in women, although there were differences based on mode of delivery, this observation is in concordance with earlier reported (Fallani et al., 2010).

When combining fatty acid profile and milk microbiota, we found specific association of fatty acid with microbial groups. Human milk fatty acids may influence the composition and activity of specific bacteria as demonstrated here. Fat intake is rapidly reflected in human milk fatty acid composition, which in turn may favor specific bacteria utilizing certain fatty acids incorporating them into their cell walls. Changes in the fatty acid profiles in microbial cell surfaces may advance or hinder their ability to colonize the infant gut. Our study suggests that *Staphylococcus* genus and other bacterial genus as *Pseudomonas* were negatively correlated with MUFA in TAG, whereas *Streptococcus* genus was negatively correlated with SAFA in TAG. In phospholipids, the levels of *Bifidobacterium* and *Lactobacillus* genera showed a negative association with MUFA and n-3 PUFA may suggest specific interactions, which need further validation. A previous study comparing gut microbiota profile of lactating women, has shown specific negative association of n-3 fatty acids with Bacteroidetes phyla and positive association with Firmicutes phyla (Carrothers et al., 2015). We could not find similar associations in this study, which could have been attributed to difference in sampling sites as our study is focused on breast milk and used geographical location as one of the major contributing factor. Also, the number of samples in each group could be one of the limiting factors in finding strong association with specific microbial groups. Regional dietary difference may also contribute affects the breast milk composition, for example due to increase consumption of n-3 PUFA (linoleic acid) containing vegetable oil products in western diet, has led to increase in linoleic acid concentration from 50 to 90% of the PUFAs (Liu et al., 2016). In another study involving 514 health lactating chinese mothers did not find correlation of dietary intake of Linoleic acid with milk higher dihomo γ-linolenic acid, arachidonic acid or docosatetraenoic level, which they proposed could be due to gene polymorphism affecting fat storage (Liu et al., 2016). SFA present in breast milk is related with carbohydrate and fats in daily diets as well as total energy intake and mobilization of adipose tissue (Krešić et al., 2013). The fatty acid composition along with specific microbial constitution might reflect directly in immune programming in infants. *Bifidobacterium, Lactobacillus, Bacteroides*, and *Clostridium spp.* have been associated with immunological functions such tolerance, mucus production, tight junction expression and T helper cell balance, which helps in intestinal barrier homeostasis (Walker and Iyengar, 2015). Also dietary intakes have shown to have strong impact on gut microbiota. Such associations should be further characterized to understand the role of mother’s nutrition and fatty acid intake on breast milk fatty acid and microbiota profiles. There is also evidence that human milk hormones are directly associated with gut microbial functional pathways implicated in infants intestinal inflammation (Lemas et al., 2016). Recent study based on early gut microbiota of infants from Finland, Estonia and Russia has shown that differences in *Bacteroides* spp. derived LPS when compared to *Escherichia coli* derived LPS has differential immunogenic roles, which could be associated with higher incidences of auto-immune diseases prevalent in Northern Europe (Vatanen et al., 2016). Such studies including ours provide new insights and may propose novel tools for dietary recommendations and nutrition counseling for breast-feeding mothers.

## Conclusion

Our results demonstrate differences in the composition of lipids and microbiota in breast milk in different geographic regions and offer a new insight to the differences in development of gut microbiota in infants in different geographic areas. The impact of mode of delivery on milk microbiota composition appears to be even more important within all geographical regions. Furthermore, our study shows distinct association between fatty acid composition and individual microbiota in breast milk, suggesting important role of lipids in shaping the microbial profile which likely one important factor influencing the infant gut microbiota development.

## Author Contributions

The work was initially designed by HK, ET, MC, KL, BY, EI, and SS. Data contributors were HK, ET, AK, JA, YZ, and MN. The manuscript was drafted by HK, MC, ET, KL, and SS edited and approved by all authors.

## Conflict of Interest Statement

The authors declare that the research was conducted in the absence of any commercial or financial relationships that could be construed as a potential conflict of interest.

## Footnotes

1 http://cgenome.net/calypso/

2 http://huttenhower.sph.harvard.edu/galaxy/
