# Supplementary material for: Distinct Patterns in Human Milk Microbiota and Fatty Acid Profiles Across Specific Geographic Locations
Source: Front Microbiol. 2016 Oct 13;7:1619. doi: 10.3389/fmicb.2016.01619 (PMC5061857; doi:10.3389/fmicb.2016.01619)
Supplement: Supplementary file 1 [file Table_1.DOCX]

Supplementary Material

**Distinct patterns in human milk microbiota and fatty acid profiles across specific geographic locations**

Himanshu Kumar,^*^ Elloise du Toit, Amruta Kulkarni, Juhani Aakko, Kaisa M. Linderborg, Yumei Zhang, Mark P. Nicol, Erika Isolauri, Baoru Yang, Maria Carmen Collado, Seppo Salminen

**Correspondence:** Himanshu Kumar, kumhim@utu.fi

# Supplementary Table Relative abundancies (%) of phospholipipids and triglycerides presented as predicted marginal means with 95% confidence intervals. Significance levels were determined by two-way analysis of variance testing the effect of country, mode of delivery and their interaction. Differences between factors were determined by Tukey HSD post-hoc comparisons. Bolded p-values: significant differences.

|  | **China** | **Finland** | **South Africa** | **Spain** | **p (Country)** | **p (delivery)** | **p (country*delivery)** |
| --- | --- | --- | --- | --- | --- | --- | --- |
| PL SAFA | 42.21 (41.00 - 43.41) | 49.76 (48.15 - 51.36) | 48.25 (46.91 - 49.60) | 47.13 (44.55 - 49.71) | < 0.001 | 0.089 | 0.61 |
| PL MUFA | 17.82 (16.43 - 19.21) | 19.27 (17.87 - 20.67) | 20.50 (18.31 - 22.70) | 19.66 (17.65 - 21.67) | 0.11 | 0.50 | **0.001** |
| PL OMEGA3 | 4.51 (3.86 - 5.16) | 4.78 (4.07 - 5.50) | 3.44 (2.64 - 4.24) | 3.74 (3.33 - 4.15) | 0.003 | 0.72 | **< 0.001** |
| PL OMEGA6 | 34.84 (34.08 - 35.60) | 25.52 (24.06 - 26.98) | 26.96 (25.02 - 28.90) | 27.54 (25.52 - 29.55) | **< 0.001** | **0.002** | **0.007** |
| PL PUFA | 39.36 (38.55 - 40.16) | 30.30 (28.85 - 31.76) | 30.40 (28.03 - 32.77) | 31.28 (29.49 - 33.06) | **< 0.001** | **< 0.001** | **< 0.001** |
| PL Others | 0.62 (0.54 - 0.70) | 0.67 (0.54 - 0.80) | 0.84 (0.68 - 1.00) | 1.94 (1.19 - 2.68) | **< 0.001** | **0.038** | **0.026** |
| TAG SAFA | 34.71 (32.99 - 36.43) | 48.22 (45.94 - 50.49) | 48.31 (45.49 - 51.13) | 46.11 (40.32 - 51.90) | **< 0.001** | 0.20 | 0.15 |
| TAG MUFA | 35.43 (33.52 - 37.34) | 36.55 (34.95 - 38.15) | 34.68 (32.66 - 36.70) | 36.63 (32.17 - 41.09) | 0.65 | 0.010 | **0.045** |
| TAG OMEGA3 | 2.71 (2.04 - 3.38) | 2.42 (2.02 - 2.82) | 1.44 (1.21 - 1.68) | 1.01 (0.85 - 1.18) | **< 0.001** | 0.25 | 0.20 |
| TAG OMEGA6 | 25.66 (23.61 - 27.71) | 10.32 (9.01 - 11.64) | 13.41 (11.93 - 14.90) | 14.67 (12.31 - 17.03) | **< 0 001** | 0.70 | 0.52 |
| TAG PUFA | 28.37 (26.18 - 30.57) | 12.74 (11.20 - 14.28) | 14.85 (13.27 - 16.44) | 15.68 (13.25 - 18.12) | **< 0.001** | 0.90 | 0.38 |
| TAG Others | 1.49 (1.34 - 1.65) | 2.49 (2.16 - 2.81) | 2.15 (1.90 - 2.41) | 1.57 (1.29 - 1.86) | **< 0.001** | 0.58 | 0.48 |
